# Building visualization skills through investigating the *Journal of the Medical Library Association* coauthorship network from 2006–2017

**DOI:** 10.5195/jmla.2020.775

**Published:** 2020-04-01

**Authors:** Rebecca Reznik-Zellen, Alexander J. Carroll, Eileen G. Harrington, Douglas James Joubert, Tyler Nix, Kristine M. Alpi

**Affiliations:** Head, Science and Engineering Library, University Libraries, University of Massachusetts, Amherst, MA, rreznikz@library.umass.edu, http://orcid.org/0000-0001-9321-8284; Librarian for Science, Technology, Engineering, and Mathematics (STEM) Research, Sarah Shannon Stevenson Science & Engineering Library, Vanderbilt University, Nashville, TN, alexander.j.carroll@Vanderbilt.Edu, http://orcid.org/0000-0003-0248-3811; Assistant Director and Health & Life Sciences Librarian, Priddy Library, Universities at Shady Grove, Rockville, MD, eharring@umd.edu, http://orcid.org/0000-0002-8964-1570; Informationist, Division of Library Services, National Institutes of Health, Bethesda, MD, douglas.joubert@nih.gov, http://orcid.org/0000-0003-4090-5587; Informationist, Taubman Health Sciences Library, University of Michigan, Ann Arbor, MI, tnix@umich.edu, http://orcid.org/0000-0002-0503-386X; University Librarian, OHSU Library, Oregon Health & Science University, Portland, OR, alpi@ohsu.edu, http://orcid.org/0000-0002-4521-3523

## Abstract

**Objective:**

The primary objective of this study was to explore different dimensions of *Journal of the Medical Library Association (JMLA)* authorship from 2006–2017. Dimensions that were evaluated using coauthorship networks and affiliation data included collaboration, geographical reach, and relationship between Medical Library Association (MLA) member and nonmember authors. A secondary objective was to analyze the practice and practical application of data science skills.

**Methods:**

A team of librarians who attended the 2017 Data Science and Visualization Institute used *JMLA* bibliographic metadata extracted from Scopus, together with select MLA membership data from 2006–2017. Data cleaning, anonymization, analysis, and visualization were done collaboratively by the team members to meet their learning objectives and to produce insights about the nature of collaborative authorship at *JMLA.*

**Results:**

Sixty-nine percent of the 1,351 *JMLA* authors from 2006–2017 were not MLA members. MLA members were more productive and collaborative, and tended to author articles together. The majority of the authoring institutions in *JMLA* are based in the United States. Global reach outside of the United States and Canada shows higher authorship in English-speaking countries (e.g., Australia, United Kingdom), as well as in Western Europe and Japan.

**Conclusions:**

MLA support of *JMLA* may benefit a wider network of health information specialists and medical professionals than is reflected in MLA membership. Conducting coauthorship network analyses can create opportunities for health sciences librarians to practice applying emerging data science and data visualization skills.

## INTRODUCTION

In response to academic and research libraries developing new data-intensive services to support twenty-first century research methods [1, 2], formal continuing education and training opportunities for librarians in data science and data visualization have proliferated [3–6]. These courses offer broad introductions to a number of topics related to data science and data visualization; however, to utilize the skills gained and retain the knowledge learned, librarians must gain experience applying these skills in real-world contexts at their local institutions [7]. Unfortunately, librarians have noted the challenge of finding opportunities at their local institutions to put these newly minted skills to use [8]. For stakeholders to ask librarians to use these skills, librarians must first raise awareness of these new skill sets by proactively creating opportunities that will demonstrate how these skills can create solutions for their stakeholders’ problems [9, 10]. Others have suggested a number of different approaches for creating these opportunities, from designing enterprise-level services to creating workshop series that offer data science training opportunities for undergraduate and graduate students [11–13].

Assisting university senior leadership, research administrators, and individual faculty with quantitative assessments of research impact has emerged as a growing area of need at many institutions and presents a unique opportunity for information specialists who are interested in applying data science and data visualization skills [14]. Coauthorship network analysis, a method for visually demonstrating research impact, can reveal the collaborative patterns and behaviors of authors at individual, institutional, or geographical levels [15, 16]. While most previously published analyses of coauthorship networks have studied collaboration patterns in a research domain or in a single institution, a handful of studies have utilized this method to identify networks in closely related conference proceedings [17] or in single journal titles [18]. Health sciences librarians at the 2017 Data Science and Visualization Institute (DSVIL) [4] discussed the possibility of generating coauthorship networks analyses based on published *Journal of the Medical Library Association (JMLA)* data.

Previous *JMLA* studies have evaluated topical content and trends [19, 20] but not coauthorship networks. The authors hypothesized that a network analysis could create insights on authorship and collaboration among contributors that would build on previous surveys of health sciences librarians’ research engagement and extend our understanding of Medical Library Association (MLA) member scholarly output [21, 22]. Furthermore, a coauthorship network analysis of *JMLA* authors could help our research team meet our individual learning objectives for using the tools and understanding the mechanisms of network analysis, while also producing insights about the nature of collaborative authorship that might be of strategic interest to MLA. To that end, our goal was to analyze *JMLA* coauthorship networks from 2006–2017 and to explore dimensions of *JMLA* authorship, investigating three specific research questions:

1. What is the extent of collaboration by *JMLA* authors?
2. Does membership in MLA influence collaboration and if so, how?
3. What is the geographic distribution of *JMLA* authorship?

## METHODS

Our research team created coauthor network visualizations of *JMLA* publications from 2006–2017 using Sci2 [23] and Gephi [24]. Sci2 and Gephi are two openly available and open source software programs that enable users to analyze and visualize networks using bibliographic datasets [25]. Because *JMLA* had a new agreement with the National Library of Medicine regarding its open access model by 2006, we chose that year as a starting point for the dataset in order to avoid authorship anomalies that might result from possible changes in the journal’s business model. Our data sources for creating this network visualization included *JMLA* bibliographic metadata drawn from Scopus and Web of Science, as well as MLA membership data.

We obtained the following membership data elements under a research data use agreement with MLA: First Name, Last Name, Library Name (if available), Institution Name, City, State, Country, MLA membership status, and Academy of Health Information Professionals (AHIP) membership status. The membership data included those who were members at any time during the 2006–2017 time frame. The only members who were not included in the data set from MLA were those from European Union countries due to changing privacy requirements that went into effect in May 2018. European authors were checked manually by name using the Search function of the member-only MLA membership lookup to assess their MLA membership status.

The study protocol was reviewed and approved by the North Carolina State University Institutional Review Board (IRB) on April 16, 2018 (IRB protocol 12862). As the name data were held confidentially by our research group through agreement with MLA, no consent was sought from any individual member in their database. No member names are shown in the analysis and visualization.

There are 2 potential fields in a Scopus record that can contain author address information: (1) the Affiliation field or (2) the Corresponding Address field. There is only 1 field in a Web of Science record that contains author address information, the C1 field, which contains the author address. We used the Scopus Affiliation field and the Web of Science C1 field to compare the records between the 2 databases. There were 20 Scopus records that had no Affiliation data and 62 Web of Science records that were missing an author address. Thus, after examining data from these fields, we chose to use the data from Scopus as the basis of the analysis because it had more complete author address information. The starting Scopus data reported 1,966 authors of 756 publications, and the MLA membership data included names and membership status information for 3,695 individuals.

Our data preparation included three overarching goals and associated work flows: joining, cleaning, and merging (Table 1). *JMLA* author data from Scopus was joined with MLA membership data to enable analysis of coauthor membership statuses. The Excel VLOOKUP function was used to join *JMLA* authors with status of membership in MLA and the academy. To ensure correct joining between names that had one or two initials, VLOOKUP was run against both last name, first initial and last name, first and second initial, and then compared. Results that differed were manually checked against the MLA data to verify identities.

**Table 1:** Summary of data preparation goals

Data work flow	Goal	Tools	Number of authors
Joining	Append MLA membership status to each author in the Scopus dataset.	Excel VLOOKUP function	1,966
Cleaning	Determine whether authors with very similar names are the same author or 2 distinct authors.	OpenRefine clustering and Excel VLOOKUP function (primary process)	1,368
		Sci2 node merging function (secondary process)	1,359
Merging	Consolidate results from the central cleaning work flow (Excel) and secondary cleaning work flow (Sci2) into one data set and resolve conflicts.	Gephi	1,351

Author data cleaning was performed to isolate and review instances of very similar author names in the *JMLA* author dataset to best account for distinct authors with similar names, authors with name variants, misspellings, and so on. Our primary data cleaning process used OpenRefine nearest neighbor clustering and Excel VLOOKUP to combine authors with the same last name and first and second initials, resulting in 1,368 unique authors.

In addition to our primary data cleaning process, we also performed an independent, secondary data cleaning process using Sci2’s data preparation tools. In this process, names with matching last name and first initial but one additional middle initial (e.g., Smith J. and Smith J.A.) were not manually checked, and the less specific name variant was merged into the more specific name variant. Names with differing middle initials (e.g., Smith J.A. and Smith J.B.), with differing first initials (e.g., Smith J.A. and Smith K.A.), and with slightly different last name spellings (e.g., Smith and Smithe) were manually checked against full-text articles to determine whether the name variations should be merged or in fact represented 2 different individuals, resulting in 1,359 unique authors.

When the Excel MLA membership data joining and the two independent data cleaning processes were complete, the data sets were merged and conflicts were resolved through manual checking, resulting in 1,360 unique authors or nodes. A node represents a single unit of analysis, in this case an author. Node merges based on the resolved dataset were performed in Gephi. A final visual check of author names found 7 authors with 1 more variant to be merged. In the process of checking these data, we discovered that 2 author names were created erroneously from a named professorship title that had been parsed by Scopus as 2 additional authors. As these 2 nodes were not associated with other papers, they were deleted along with their associated edges, their connections to other authors, in this case coauthorships. The final count was 1,351 unique author nodes.

Names and institutions were de-identified by replacing them with unique identifiers before creating the visualization of *JMLA* authorship in Gephi. We used Research Randomizer [26] to generate 1,351 unique random numbers to assign to the nodes. Once the crosswalk from author name to random unique identifier was made, the author names were replaced with the unique random number so that the data set was de-identified. The network was then visualized using Gephi.

We compiled the geographic attributes of *JMLA* authorship by first manually counting in the Scopus *JMLA* data the number of institutions in each state or province in the United States and Canada and the number of institutions in each country worldwide with at least one author. We conducted these counts at the institutional level, rather than at the individual department or library level in a larger institution. The only exception to this was that any hospital or clinic associated with a university was counted as a separate institution. For example, authors from various branch libraries at an institution were counted only once in the total. Individuals listed as consultants or independent researchers were counted as single institutions. The counts were divided into the following categories: higher education institutions, hospitals (which included clinics), organizations (governmental and nongovernmental agencies and companies), and independent consultants or researchers.

We mapped the resulting data set of the distribution of the total number of institutions in the United States and Canada with at least one *JMLA* author using ArcGIS 10.3 and used publicly available shapefiles to create the base maps [27, 28]. Since higher education institutions constituted the largest number of institutions with *JMLA* authors, we normalized that data against the total number of higher education institutions in each state and province in the United States and Canada [29, 30]. We then created a map of these data in the same manner as the first map.

We also created a dot map of the total number of institutions using ArcGIS Online. This map did not include dots for those states or provinces that did not have any institutions with a *JMLA* author. The map included pop-ups that showed each state or province’s number of institutions with at least one *JMLA* author and its percentage of combined US and Canadian institutions with at least one *JMLA* author. We used these same procedures in ArcGIS Online to map the number of institutions by country and the number of higher education institutions by state or province as a percentage of the total number of higher education institutions.

## RESULTS

**Figure 1:**
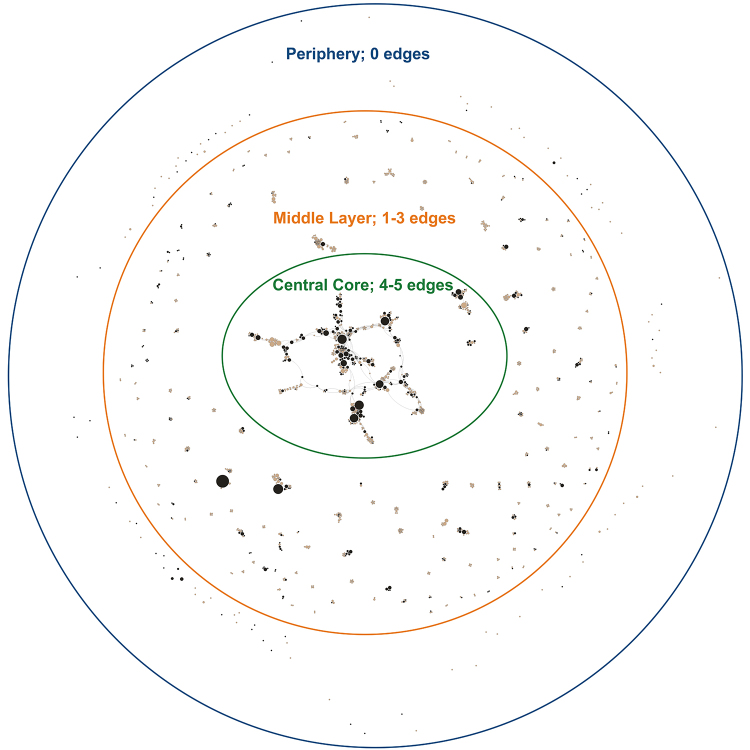
Coauthorship network of all *Journal of the Medical Library Association (JMLA)* authors 2006–2017 Nodes represent individual authors, sized by total number of published papers and color-coded for MLA membership status, with black indicating MLA members and tan indicating non-MLA members. Edges represent connectivity between authors. This network was created using Gephi; node placement was determined by the ForceAtlas2 algorithm.

Our final data set for network visualizations contained de-identified data for the 1,351 unique authors in the *JMLA* data set, including their random identifiers, their total number of publications, MLA and academy membership status, and coauthorship information. Most authors (69%, n=926) were not MLA members. Of the 31% (n=425) of authors who were MLA members, 11% (n=143) were members of the Academy of Health Information Professionals at some point from 2006–2017. In addressing 2 of our research questions, we designed a visualization that depicted the overall collaboration network in *JMLA* and MLA membership status for individual authors. Rather than depicting these 2 variables (i.e., collaboration and membership) separately, we created a single composite visualization that mapped both variables (Figure 1).

Figure 1 addresses our research questions about the extent of *JMLA* authorship and the influence of MLA membership on collaboration. Figure 1 was created in Gephi, using the ForceAtlas2 algorithm. ForceAtlas2 is a force-directed placement algorithm that functions so that nodes repel one another, but connected nodes are drawn together spatially [31]. Therefore, this network features concentric rings (a periphery, a middle layer, and a central core) that show increased collaboration toward the center. The distinction between rings is determined by edge weight (0–5): the periphery contains nodes with 0 edges; the middle layer contains nodes with edge weights of 1–3; and the central core includes nodes with edge weights of 4 and 5. The nodes are color-coded to indicate MLA membership status, making the relationship between MLA-member authors and non-MLA member authors visible. The nodes are sized by total number of published papers to illustrate productivity.

The majority of authors published only 1 paper in *JMLA* between 2006 and 2017 (1,033 of 1,351, 76%), and they are distributed throughout the network. A smaller number of 2-paper authors were also distributed around the visualization (191, 14%). Included in the middle layer and central core were the 5% of authors who published 3 papers in *JMLA*, the 1% who published 5 papers, and the less than 1% of authors who published more than 10 papers. MLA members as a group authored fewer *JMLA* papers than non-MLA members as a group (42% and 58% of papers, respectively); however, MLA members were the more productive authors in this data set. To compare, 60% of MLA members were single-paper authors (19% of the total), whereas 84% of non-MLA members were single-paper authors (57% of the total). Although some non-MLA members authored multiple papers in *JMLA*, MLA members constituted all nodes with the highest number of authored works in this data set (8–17 of a maximum 17).

Most authors collaborated on only 1 paper in *JMLA* during 2006–2017 (94%). Figure 1’s periphery shows 1- and 2-paper authors with no collaboration between authors. Collaborations of 2 to 5 or more authors make up the majority of the middle layer but with disconnected clusters in this layer. While authors who published more than 1 paper were more likely to have collaborated on more than 1 paper, there were a few notable exceptions. In particular, there were some authors who had relatively high levels of productivity but a low number of collaborations. We hypothesized that these nodes likely were editors of the journal, as we did not exclude items from the data based on document type.

MLA member and non-MLA member authors were well distributed throughout the visualization. Although there was little collaboration among authors in the middle layer of the network, it was common to have at least 1 MLA member in clusters of papers with 2 or more authors, and MLA members tended to coauthor together. The central core was composed of both MLA and non-MLA members. MLA members were more productive and connected than non-MLA members, although there were also some non-MLA members with more than 1 or 2 papers in the core. Most of the connecting nodes in the central core were MLA members or were node clusters that included an MLA member, although there were a few connecting nodes that were non-MLA members. MLA members who were highly productive were either directly connected or were indirectly connected to one another through authors with lower productivity who were either MLA members or non-MLA members.

**Figure 2:**
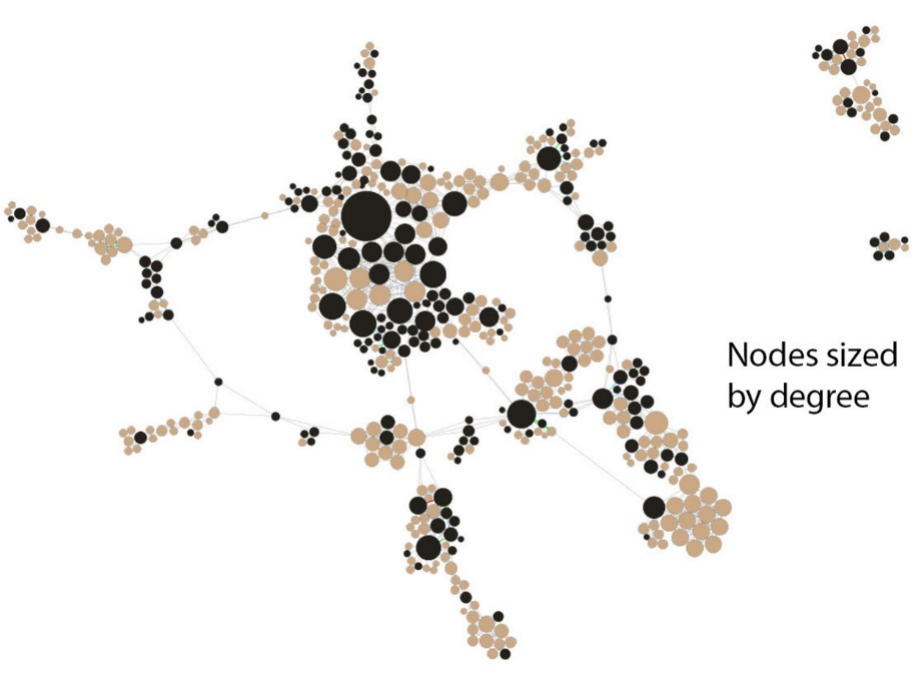
Central core of *JMLA* coauthorship network showing collaborative author relationships Nodes sized by degree (number of collaborative author relationships) and color-coded by MLA member status, with black indicating MLA members and tan indicating non-MLA members.

**Figure 3:**
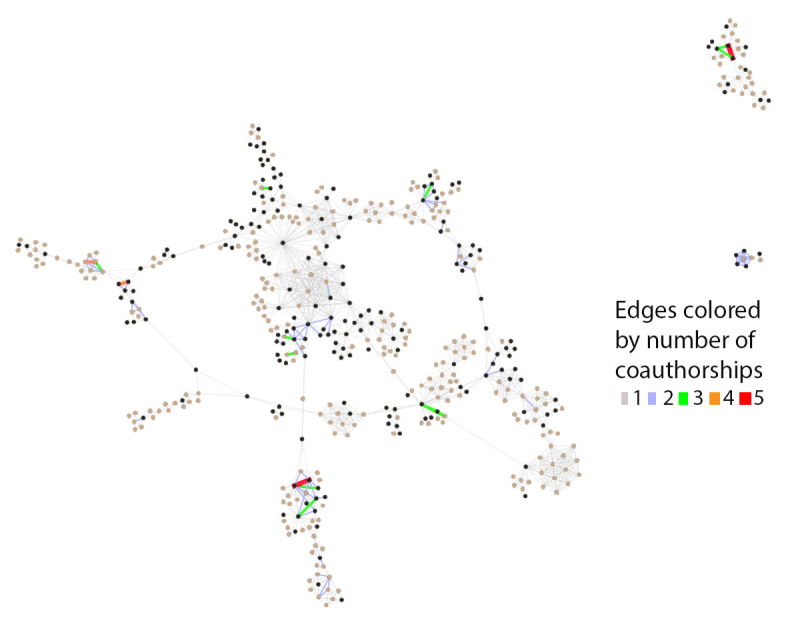
Central core of *JMLA* coauthorship network showing number of coauthorships Nodes sized uniformly and color-coded by MLA member status, with black indicating MLA members and tan indicating non-MLA members. Edges are color-coded for number of coauthorships.

Two views of the inner core demonstrate how distinctions can be made when highlighting different data elements of the network in Gephi. In Figure 2, we sized the nodes to represent degree of connectedness, rather than productivity. This view visually highlighted the authors who had more connections in the network. In Figure 3, we sized the nodes uniformly, which increased the visibility of the edges between each node. These edges represented connections between nodes, in this case number of coauthorships. The range of coauthorships between nodes in the central core was 1–5. In comparing the two figures, Figure 2 demonstrates authors who collaborate frequently with many different authors, whereas Figure 3 demonstrates authors who collaborate frequently with the same coauthors.

In addition to describing the visual appearance of the network, we used Gephi to calculate basic network statistics to quantitatively describe the network with the goal of allowing the *JMLA* network to be compared statistically with other authorship networks (e.g., Börner et al. [16], Liu et al. [17], Agarwall et al. [32]). We chose three statistics from those highlighted at DSVIL: two that described the network (i.e., average degree and modularity) and one that described the author nodes (i.e., clustering coefficient). The average degree of a node is the number of edges connected to it, in this case the number of connections with other authors.

For the *JMLA* authorship network, the average degree was 4.3, which meant that the average author was connected with approximately 4 other *JMLA* authors. Modularity indicates author interconnectivity by detecting author communities or densely connected nodes. Modularity measures the structure of the network by dividing it into modules that represent communities. The modularity for this network based on 756 papers was 0.9, with 302 communities of authors. The modularity statistic itself allowed comparison across networks of different sizes, while the number of communities was comparable only for similar volumes of publications. Finally, the clustering coefficient is a measure of the degree to which nodes in a graph tend to cluster (in this case, coauthor) together. The average clustering coefficient, the mean value of individual coefficients, for this network was 0.9. This showed that there was a fairly high tendency toward clustering in this network (going toward 1), which could be interpreted in a coauthor network as the authors in the network being likely to collaboratively author in the future.

**Figure 4:**
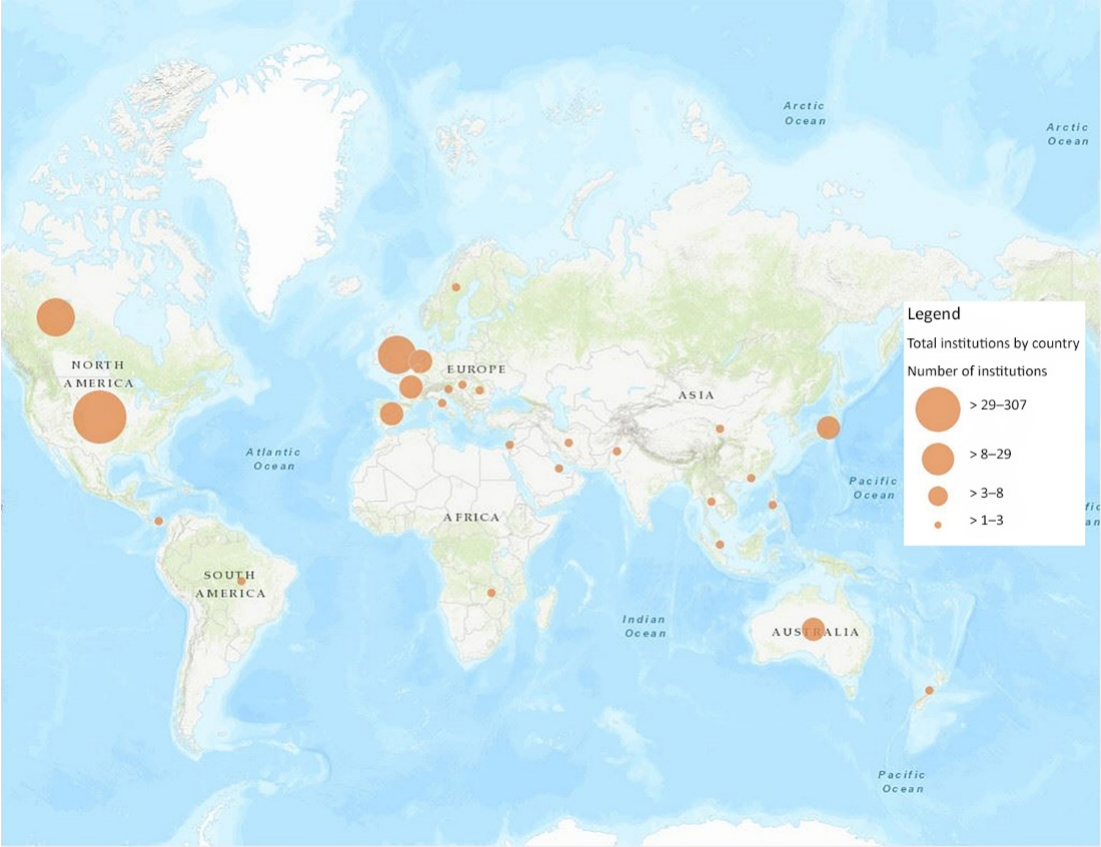
Global distribution of *JMLA* authorship showing geographic locations of institutions that had at least one *JMLA* author from 2006–2017 Created using ArcGIS Online. An interactive version of the map is available online [33].

Our final research question led us to examine the geographic distribution of *JMLA* authorship. Figure 4 shows a screenshot of a map of the global distribution of institutions with *JMLA* authors from 2006–2017, which was created using ArcGIS Online. An interactive version of this map is available online [33]. As expected, the majority (n=287, 73%) of the authoring institutions were based in the United States. Global engagement outside of the United States and Canada showed higher authorship in English-speaking countries (e.g., Australia, United Kingdom), as well as in Western Europe and Japan. There were very few authoring institutions in Africa, Eastern Europe, or Latin America.

**Figure 5:**
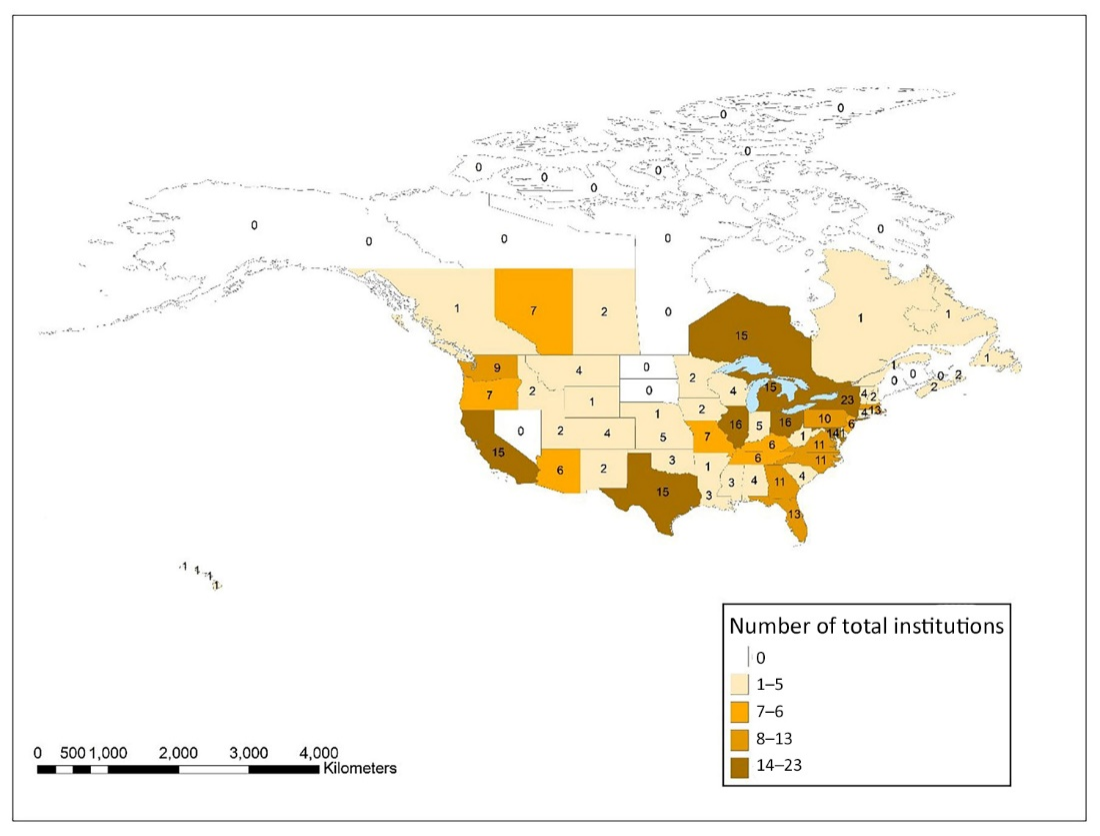
Distribution of the total number of all institution types with at least one *JMLA* author in the United States and Canada An interactive version of the map is available online [34].

Figure 5 shows the total number of all types of institutions in each state, province, and territory in the United States and Canada that had at least 1 author in *JMLA* from 2006–2017. An interactive map using these same data was also created using ArcGIS Online and is available online [34]. The state with the most overall institutions with at least 1 *JMLA* author was New York with 23, followed by Illinois and Ohio, both at 16, and California, Michigan, Ontario, and Texas, all with 15. These 7 states represented 34% of all institutions in the United States and Canada with authorship in *JMLA*. Eleven states, provinces, or territories did not have any institutions with *JMLA* authors: Alaska, Maine, Manitoba, Nevada, New Brunswick, North Dakota, Northwest Territories, Nunavut, Prince Edward Island, South Dakota, and Yukon.

**Figure 6:**
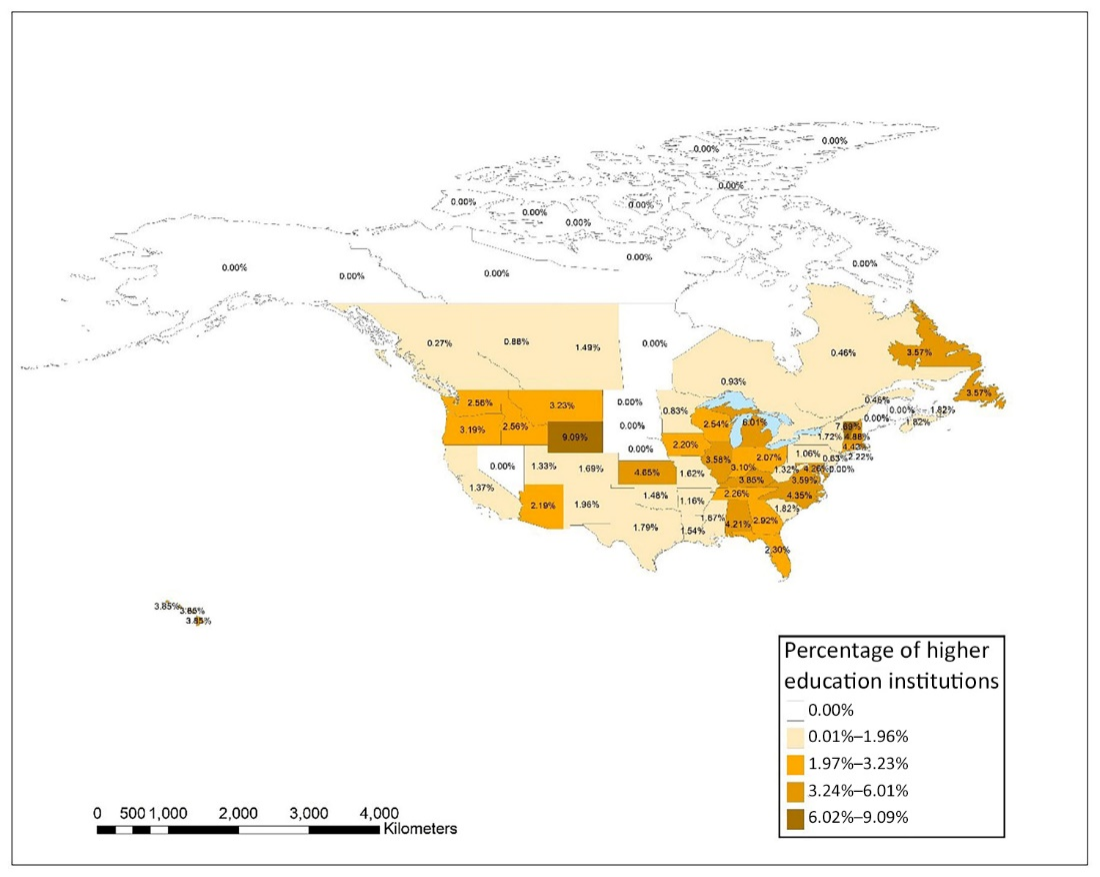
Distribution of higher education institutions in the United States and Canada with at least one JMLA author as a percentage of the total number of higher education institutions in each state or province An interactive version of the map is available online [35].

Figure 6 shows the distribution of higher education institutions in each state and province in the United States and Canada that had at least 1 author in *JMLA* from 2006–2017 as a percentage of the total number of higher education institutions. An interactive map using these same data was also created using ArcGIS Online and is available online [35]. The top 5 states, provinces, or territories with the largest percentage of authors from higher education institutions were Wyoming (9.1%), Washington, DC (8.7%), Vermont (7.7%), Michigan (6.0%), and New Hampshire (4.9%). There was not a clear pattern of states with a larger number of higher education institutions having higher rates of publication in *JMLA*. In fact, with the exception of Michigan, the other states in the top 5 had only 1 or 2 institutions with *JMLA* authorship representation, but they also had lower overall numbers of higher education institutions.

## DISCUSSION

We undertook this project as both a learning exercise and an opportunity to advance our knowledge about authorship patterns in health sciences librarianship. From our learning perspective, the methods described reflected the approaches we took, given the knowledgebase and time available among our members. This learner-centered approach to conducting this research project had benefits and drawbacks. In some cases, we used familiar tools and approaches that might or might not have been the most efficient solutions. For example, we investigated and experimented with using OpenRefine [36] for data cleaning; however, we ultimately elected to perform multiple rounds of data cleaning using tools that we had more experience with and greater confidence in the outputs. This approach of using parallel data cleaning work flows in turn resulted in different yields, which required an extra step to resolve conflicting yields.

In other cases, we pushed ourselves to try new tools and approaches, which then increased the time required to accomplish objectives. For example, to learn more about Gephi, we explored different layouts before choosing ForceAtlas2 as the best way to represent the *JMLA* network. A researcher with more experience might have been more efficient if learning were not a primary objective of the exercise. We also limited ourselves to a few basic network statistics that we had learned about at DSVIL or were reported in relevant literature [16, 17, 32]. We did not expand to the many network statistics that could have been calculated, as which statistics are most useful for describing coauthorship networks is an open research area [16].

Similarly, while we recognized that we could have obtained a partial answer to many of our research questions with tabular data from Scopus or Web of Science, we sought to use this project to learn how to visualize these data and consider how the visualizations could create additional insights beyond the data available from Scopus or Web of Science. In our opinion, these visualizations more effectively demonstrated the relationships between MLA and non-MLA member authors as well as depicted the prevalence of non-MLA member authorship. Figures 1, 2, and 3 visually demonstrated the distribution of high and low productivity authors throughout the network as well as the role played by non-MLA members in the network; these insights would not have been gained through the data alone. Likewise, the geographic distributions in Figures 4, 5, and 6 enhanced the impact of the data, revealing stark patterns that were not as readily apparent when looking at a tabular list of the number of institutions from each country, state, or province with *JMLA* authorship. This was particularly useful for creating insights into global patterns of *JMLA* authorship.

We had thought of the three research questions that we addressed in the results separately and did not have an a priori idea of how they might be interrelated. *JMLA* authorship comprised more non-MLA members than MLA members, whereas a small number of MLA members were the most productive and collaborative authors. However, lower-productivity MLA members and non-MLA members still played an important role in connecting the authorship network.

Our findings generally aligned with the results from similar studies performed by Liu et al. [17] and Zare-Farashbandi et al. [18]. These studies, in their single title coauthorship studies, also found that a small number of authors were the most prolific and collaborative, resulting in similar network statistics. Using the metric of mean distance, which is the average number of edges on the shortest path connecting pairs of nodes, our network had a mean distance of 4.3, while Liu et al. and Zare-Farashbandi et al. calculated mean distances of 3.0 and 4.0, respectively. We found similar alignment with clustering coefficients: Liu et al. and Zare-Farashbandi et al. reported clustering coefficients of 0.9 and 0.8, compared to our network’s clustering coefficient of 0.9.

While the majority of authors with more than 2 publications were MLA members, we could not assume that membership in MLA was the reason for publishing more in *JMLA*, though there might be aspects of membership that facilitated collaboration on *JMLA* articles. The high rate (69%) of publications in *JMLA* by non-MLA members suggested that publishing in *JMLA* was accessible and attractive to authors from institutions or countries with no or low MLA membership. While the geographic distribution of MLA members might be associated with or influenced by the geographic distribution of *JMLA* authorship, this was not a question that we specifically addressed. However, one implication of this finding was that MLA member support for editing, peer reviewing, and hosting *JMLA* might benefit a broader range of health information specialists and medical professionals than chose to join the association. It bears noting that given the data sets used for this study, there was no way to determine if an author was an MLA member in the same year that their paper was published.

The intensive data cleaning methods utilized in this study reflected the complexity and lack of standardization of institutional data as published in *JMLA* and as maintained in the MLA member database; however, many of these difficulties created by author name ambiguity and inconsistent affiliation data might be addressed in the near future if uptake of interoperable researcher identifiers such as ORCID increase among *JMLA* authors as a community [37].

The limitations created by these data cleaning work flows, as well as privacy concerns, impacted the fidelity of results presented in these visualizations. For example, to protect the privacy of individual contributors, we presented geographic data at the state level and examined only institutional-level contribution to *JMLA* authorship. Although we were able to normalize the geographic data for authors from higher education institutions, the baseline data of higher education institutions included a broad range of postsecondary institutions, many of which likely did not have any health-related programs. This might skew the data somewhat. Furthermore, these visualizations represented works that successfully navigated through the necessary editorial and peer-review processes to be published in the journal. We do not know whether the absence of *JMLA* publications from various parts of the world represented an absence of author interest in publishing in *JMLA* or if author submissions from those countries were not successful in being accepted for publication.

Researchers from six institutions in five states across three time zones collaborated on this study. Therefore, investigating these research questions via an interinstitutional project created additional challenges that required coordinating shared work and contributions among our diverse team.

Our team was able to conduct this research entirely remotely using online meeting rooms and shared document storage. However, several members moved in and out of contributing due to changes in their situations as the timeline for this project expanded considerably from our initial plan of a twelve-month project completed by 2018 to a twenty-four-month project finished in 2019. Scheduling demands played a significant role in this expansion. In part to address time constraints, we limited our scope and decided not to examine how institutional affiliation affected coauthorship.

We had also intended to look at trends in authorship over time by slicing the visualizations by year or year ranges. These time intervals could be created by separating the authorship data initially into intervals and then following the same process that we used with the authors as the unit of analysis. Alternatively, an interested research team could create a data set focused on the publications as the unit of analysis and slice it by years without focusing on authorship. Similarly, future researchers could also consider the impact of membership in the Academy of Health Information Professionals on coauthorship networks in *JMLA*. While this study did not include any network visualizations using academy data, we collected these data, so our data set could be reused to facilitate investigating this question [38].

The challenges of interinstitutional collaboration were offset by the benefits of learning from each other and having peers who cared about reaching the outcome, which motivated us to keep going with the project.

We provide these visualizations and this analysis to offer our insight into the authorship patterns of papers selected for publication in *JMLA* from 2006–2017. We hope that the *JMLA* leadership, the MLA organization, and the health sciences librarian profession find them useful in facilitating discussions about encouraging and expanding authorship. Further, we suggest that this type of data analysis project is not only useful for librarian knowledge building related to data sciences, but is also foundational for building science, technology, engineering, and math (STEM) and health sciences support programs for the communities that we serve.

## DATA AVAILABILITY STATEMENT

All data for this project that we are authorized to share are publicly available from an Open Science Framework (OSF) project page [38]. Available datasets in this OSF project page include: the Gephi network project and its tables of de-identified nodes and edge data associated with this article; the author name disambiguations that were generated as part of the research [39]; and the .csv files of the geographic distribution of *JMLA* authorship that were used to make the maps included in this article [40, 41]. We did not seek permission to make the citation and author affiliation data from Scopus used for this analysis available, but these raw data could also be downloaded from PubMed. MLA membership data were obtained through a data use agreement with MLA that prohibits data sharing; contact MLA for information about MLA membership data access.

## 

**Rebecca Reznik-Zellen**, rreznikz@library.umass.edu, http://orcid.org/0000-0001-9321-8284, Head, Science and Engineering Library, University Libraries, University of Massachusetts, Amherst, MA

Alexander J. Carroll, AHIP*, alexander.j.carroll@Vanderbilt.Edu, http://orcid.org/0000-0003-0248-3811, Librarian for Science, Technology, Engineering, and Mathematics (STEM) Research, Sarah Shannon Stevenson Science & Engineering Library, Vanderbilt University, Nashville, TN

**Eileen G. Harrington**, eharring@umd.edu, http://orcid.org/0000-0002-8964-1570, Assistant Director and Health & Life Sciences Librarian, Priddy Library, Universities at Shady Grove, Rockville, MD

**Douglas James Joubert**, douglas.joubert@nih.gov, http://orcid.org/0000-0003-4090-5587, Informationist, Division of Library Services, National Institutes of Health, Bethesda, MD

**Tyler Nix**, tnix@umich.edu, http://orcid.org/0000-0002-0503-386X, Informationist, Taubman Health Sciences Library, University of Michigan, Ann Arbor, MI

**Kristine M. Alpi, AHIP**, alpi@ohsu.edu, http://orcid.org/0000-0002-4521-3523, University Librarian, OHSU Library, Oregon Health & Science University, Portland, OR
